# HPLC profiling, antioxidant and in vivo anti-inflammatory activity of the ethanol extract of *Syzygium jambos* available in Bangladesh

**DOI:** 10.1186/s13104-016-2000-z

**Published:** 2016-03-28

**Authors:** Hemayet Hossain, Shaikh Emdadur Rahman, Proity Nayeeb Akbar, Tanzir Ahmed Khan, Md. Mahfuzur Rahman, Ismet Ara Jahan

**Affiliations:** Chemical Research Division, BCSIR Laboratories, Bangladesh Council of Scientific and Industrial Research, Dr. Qudrat-E-Khuda Road, Dhaka, 1205 Bangladesh; Pharmacy Discipline, Life Science School, Khulna University, Khulna, 9208 Bangladesh; Institute of Food Science and Technology, Bangladesh Council of Scientific and Industrial Research, Dr. Qudrat-E-Khuda Road, Dhaka, 1205 Bangladesh

**Keywords:** *Syzygium jambos*, HPLC, Catechin hydrate, ABTS, Reducing power, Carrageenan

## Abstract

**Background:**

*Syzygium jambos* has been used as a traditional medicine for the treatment of inflammatory diseases in Bangladesh. The study investigates the high performance liquid chromatography (HPLC) profiling of phenolic compounds, and evaluates the antioxidant and anti-inflammatory activities of ethanol extract of *S. jambos* available in Bangladesh.

**Methods:**

The extract was subjected to HPLC for the identification and quantification of the major bioactive polyphenols present in *S. jambos*. Antioxidant activity was determined using 2, 2′-azino bis-3-ethylbenzothiazoline-6-sulfonic acid (ABTS) radical scavenging, reducing power assay, total antioxidant capacity, total phenolic and flavonoid content. Furthermore, the anti-inflammatory effect of the extract in rats for two different test models: carrageenan and histamine-induced paw edema was inspected.

**Results:**

High levels of catechin hydrate and rutin hydrate (99.00 and 79.20 mg/100 g extract, respectively) and moderate amounts of ellagic acid and quercetin (59.40 and 69.30 mg/100 g extract, respectively) were quantified in HPLC. Catechin hydrate from this plant extract was determined for the first time through HPLC. For ABTS scavenging assay, the median inhibition concentration (IC_50_) value of *S. jambos* was 57.80 µg/ml, which was significant to that of ascorbic acid (12.01 µg/ml). The maximum absorbance for reducing power assay was found to be 0.4934. The total antioxidant capacity, phenolic and flavonoid contents were calculated to be 628.50 mg/g of ascorbic acid, 230.82 mg/g of gallic acid and 11.84 mg/g of quercetin equivalent, respectively. At a dose of 400 mg/kg, a significant acute anti-inflammatory activity (P < 0.01) was observed in rats for both the test models with a reduction in the paw volume of 58.04 and 53.95 %, in comparison to those of indomethacin (62.94 and 65.79 %), respectively.

**Conclusions:**

The results suggest that the phenolic and flavonoid compounds are responsible for acute anti-inflammatory and antioxidant activities of *S. jambos*.

## Background

*Syzygium jambos* (L.) Alston (Myrtaceae) is a medicinal plant widely used in sub-Saharan Africa, but is also native to Malaysia, Nepal, Guatemala and Bangladesh. The bark of this plant is generally required for the treatment of pernicious attack, diarrhoea, amenorrhea and abdominal pain [1, 2]. While investigating fruit volatiles and sugar compounds the chemico-pharmacological properties of the leaf of *S. jambos* were studied [3, 4]. The anti-diabetic activity of the leaves of *S. jambos* has been examined [5]. The hydro-alcoholic and ethyl acetate extracts of the leaves of *S. jambos* showed anti-nociceptive and anti-inflammatory activity [6, 7]. Previous studies reported that ethanol extract of the leaves of *S. jambos* showed antiviral activity on type I herpes simplex and replication inhibition of vesicular stomatitis virus [8]. Several flavonoid compounds have been isolated from *S. jambos*, among which quercetin 3-O-b-d-xylopyranosyl (1-2) a-l-rhamnopyranosides and myricetin are considered significant [9]. It was recently revealed that the methanol fraction of the leaves of *S. jambos* contained ellagic acid derivatives like 3, 3, 4 %-tri-O-methylellagic acid and 3, 3, 4 %-tri-O-methylellagic acid-4-O-b-d-glucopyranoside [10]. Moreover, several ellagitannins like casuarinin, pedunculagin, strictinin, tellimagrandin I, casuarictin and tellimagrandin II have also been reported [11].

Hence, in this experiment, we attempted to investigate the HPLC profiling of bioactive polyphenolic compounds, and evaluate the antioxidant and in vivo anti-inflammatory activities of ethanol extract of the leaves of *S. jambos* growing in Bangladesh.

## Methods

### Plant material

Leaves of *S. jambos* were collected from Khulna, Bangladesh during December 2012 and identified by Bangladesh National Herbarium, Mirpur, Dhaka (Accession no: DACB 36608). The leaves were properly washed, shade dried, and powdered. The sample was then saved in an airtight container until extraction.

### Extraction

The powdered plant materials were extracted in an orbital shaker with 95 % ethanol for 7 days at room temperature to obtain ethanol extract of *S. jambos*. The extract was initially filtered in a cotton plug to get rid of the plant debris, and next through Whatman filter paper no. 1. The solvent was removed in a rotary vacuum evaporator (R-215, Buchi, Switzerland) under reduced pressure. The plant yielded a 6.29 % extract of the dried plant material, which was stored for further analysis.

### Test animals

For the screening of in vivo anti-inflammatory activity, male rats of wister strain weighing 179–205 g were used. The animals were housed under standard laboratory condition at the Pharmacology Laboratory of BCSIR, Chittagong at 25 ± 1 °C and 12/12 h light/dark cycle. The rats were given a balanced diet and water ad libitum. All experimental protocols were in compliance with Bangladesh council of scientific and industrial research (BCSIR) ethics committee on research in animals as well as internationally accepted principles for laboratory animal use and care.

### Chemicals and reagents

ABTS, gallic acid (GA), ascorbic acid, vanillic acid (VA), caffeic acid (CA), rutin hydrate (RH), (−)-epicatechin (EC), (+)-catechin hydrate (CH), (PCA), ellagic acid (EA), quercetin (QU), *p*-coumaric acid, folin-ciocalteu’s phenol reagent, indomethacin, and histamine phosphate were purchased from Sigma–Aldrich (St. Louis, MO, USA). Met hanol (HPLC), acetic acid (HPLC), acetonitrile (HPLC), ethanol, phosphate buffer (pH 6.6), trichloroacetic acid (TCA), potassium ferricyanide [K_3_Fe(CN)_6_], sodium phosphate, ferric chloride (FeCl_3_), tween 80, sodium carbonate and ammonium molybdate were of analytical grade and purchased from Merck (Darmstadt, Germany).

### Quantification of polyphenols in *S. jambos* by HPLC

Chromatographic analysis was performed on an HPLC system model Thermo Scientific DionexUltiMate 3000 Rapid Separation LC systems (RSLC) from Thermo Fisher Scientific Inc., MA, USA. They were equipped with a diode array detector (DAD: 3000RS), quaternary pump system (LPG: 3400RS) and Ultimate 3000RS autosamplier (WPS: 3000). The system was controlled by Version 6.80 RS 10 DionixChromeleon software. Acclaim^®^ C18 (4.6 × 250 mm; 5 µm) column from Dionix, USA was used for the chromatographic separation of polyphenols that was maintained at 30 °C using a column compartment (TCC: 3000).

### Chromatographic conditions

The phenolic composition of *S. jambos* was determined by HPLC using a previously described method [12, 13]. The mobile phase consisted of acetonitrile (solvent A), acetic acid solution pH 3.0 (solvent B), and methanol (solvent C). The system was run at a gradient elution program, i.e., 0 min at 5 %A/95 %B, 10 min at 10 %A/80 %B/10 %C, 20 min at 20 %A/60 %B/20 %C and 30 min at 100 %A. The flow rate was 1 ml/min and the injection volume was 20 µl. For DAD detection, the wavelength program was set right to monitor phenolic compounds at their respective maximum absorbance wavelengths as follows: λ 280 nm held for 18.0 min, changed to λ 320 nm and held for 6 min, and finally changed to λ 380 nm and held for the rest of the analysis. The DAD was set at an acquisition range from 200 to 700 nm. The detection and quantification of GA, CH, VA, CA, and EC was carried out at 280 nm, of PCA, RH, and EA at 320 nm, and of QU at 380 nm, respectively.

### Standard and sample preparation

Standard stock solutions (100 µg/ml) of phenolic compounds were prepared in ethanol. The standard solutions were prepared by further diluting the standard stock solutions in ethanol to make solutions of 20 µg/ml for each of the polyphenols except caffeic acid that was made up to 8 µg/ml and quercetin that was prepared to 6 µg/ml. All solutions were stored in the dark at 5 °C.

The calibration curves of the standards were prepared by serial dilution of the standard stock solutions (five set) with ethanol to yield 1.25–20 µg/ml for GA, CH, V A, EC, PCA, RH, EA; 0.5–8.0 µg/ml for CA, and 0.375–6.0 µg/ml for QU. The calibration curves were drawn from the chromatograms as peak area versus concentration of standard.

A solution of *S. jambos* at a concentration of 5 mg/ml was prepared in ethanol by mixing for 30 min. The samples were stored at low temperature in the dark (5 °C). Spiking of the solution samples was done with phenolic standards to identify the individual polyphenols. Before HPLC analysis was carried out, all solutions (mixed standards, samples, and spiked solutions) were filtered through 0.20 µm PTFE syringe filter (Sartorius, Germany) and degassed in an ultrasonic bath (Hwashin, Korea) for 15 min.

### Antioxidant activities

#### ABTS radical scavenging activity test

ABTS radical scavenging was determined using the method of Fan and his co-workers [14] with some modifications. 7 mM ABTS solution and 2.45 mM potassium persulfate were reacted to obtain ABTS radical cation, which was then left to stand for 16 h in a dark place at room temperature. Just before use, the ABTS solution was diluted with ethanol and an absorbance of 0.70 ± 0.02 was measured at 734 nm. 1 ml of the sample (10–250 µg/ml) was mixed properly with 1 ml of the diluted ABTS solution. The reaction mixture was allowed to stand at room temperature for 6 min before final absorbance was read at 734 nm. The ABTS scavenging activity was calculated as follows:

$${\rm ABTS}\,{\rm scavenging}\,{\rm effect} = {\rm I}\, (\%) = (A_{{\rm o}}^{} -{\rm A}_{{\rm s}} / {\rm A}_{{\rm o}} ) \times 100$$

where, A_o_ = Absorbance of blank and A_s_ = Absorbance of sample

#### Reducing power assay

The reducing power of *S. jambos* was studied using the method of Shubhra et al. and Dehpour et al. [15, 16]. Different concentrations of the extract (10–250 µg/ml) were mixed with 1 ml ethanol, 2.5 ml potassium ferricyanide [K_3_Fe(CN)_6_] (1 %) and 2.5 ml phosphate buffer (0.2 M, pH 6.6). The mixture was incubated at 50 °C for 20 min before adding a 10 % solution of trichloroacetic acid (2.5 ml) to it. The solution was further centrifuged at 3000 rpm for 10 min. The top layer of the mixture (2.5 ml) was carefully separated and reacted with 2.5 ml distilled water and 0.5 ml of 0.1 % FeCl_3_. The absorbance was measured at 700 nm. Ascorbic acid was used as the standard reference compound.

#### Total antioxidant capacity

The total antioxidant capacity was measured by the spectrophotometeric method of Prieto et al. [17]. Different concentrations of the ethanol extract were mixed with 1 ml of reagent solution, which was prepared by reacting 0.6 M H_2_SO_4_, 28 mM sodium phosphate and 4 mM ammonium molybdate. The tubes were incubated at 95 °C for 90 min. The mixture was cooled to room temperature before reading the absorbance at 695 nm against a blank sample. Ascorbic acid equivalents were calculated using the standard graph for ascorbic acid. The experiment was performed in triplicates and values were expressed as equivalents of ascorbic acid in mg per gram of extract.

#### Total phenolic content

The modified Folin-Ciocaltu method [18, 19] was followed to determine the total phenolic content of the extract. 0.5 ml of the extract (1 mg/ml) was mixed with 5 ml Folin-Ciocaltu reagent (1:10 v/v distilled water) and 4 ml (75 g/l) of sodium carbonate. The mixture was mixed and left to stand for color development at 40 °C for 30 min before reading the absorbance at 765 nm. The total phenolic content was calculated as mg of gallic acid equivalent per g using the equation obtained from the standard gallic acid calibration curve, y = 6.993x + 0.0379, R^2^ = 0.9995.

#### Total flavonoid content

Aluminium chloride colorimetric method was used for the determination of total flavonoid content [20]. 2.5 ml of aluminium chloride reagent (133 mg aluminium chloride and 400 mg sodium acetate in 100 ml of de-ionized water) was reacted with the ethanol extract (5 ml, 1 mg/ml). The mixture was left to stand for 30 min at room temperature before recording the absorbance of the reaction mixture at 430 nm. The total flavonoid content was calculated as mg of quercetin equivalent per g using the equation obtained from quercetin calibration curve, y = 6.2548x + 0.0925; R^2^ = 0.998.

### Anti-inflammatory activity

#### Carrageenan-induced oedema test

Carrageenan induced rat hind paw edema was used as the animal model to determine acute inflammation, according to the method of Lanhers and his co-workers [21]. The rats were divided into four groups (five rats per group). Group I (control) was given 1 % tween 80 in normal saline (10 ml/kg), while Group II (positive control) received 10 mg/kg b.w. of indomethacin orally. Group III and IV received 200 and 400 mg/kg b.w. of the *S. jambos* extract orally, respectively. Acute inflammation was induced in all the four groups by sub plantar injection of 0.1 ml of its suspension of carrageenan with 1 % tween 80 in normal saline in the right paw of the rats, 1 h after the oral administration of the tested materials. The paw volume was measured with a micrometer screw gause at 1-h interval after the administration of the drug and the extract. The percentage inhibition of inflammatory effect of the extract was calculated using the following expression:

$${\text{Percentage inhibition of inflammation}} = {\text{ }}[({\text{V}}_{{\text{c}}} - {\text{V}}_{{\text{t}}} )/{\text{V}}_{{\text{c}}} ] \times 100$$

where V _c_ is the average degree of inflammation by the control group and V_t_ is the average degree of inflammation by the test group.

#### Histamine-induced oedema test

Using the method of Perianayagam and his co-workers [22] the paw oedema was produced by sub-plantar administration of 0.1 % freshly prepared solution of histamine into the right hind paw of the rats. Twenty rats were divided into four groups of five animals each. Group I (control) was supplied with 1 % tween 80 in normal saline (10 ml/kg). Group II (positive control) received 10 mg/kg b.w. of indomethacin orally. Group III and IV were given 200 and 400 mg/kg b.w. of *S. jambos* orally, respectively. Acute inflammation was produced in all the four groups by sub plantar injection of 0.1 ml of histamine with 1 % tween 80 in normal saline in the right hind paw of the rats, 1 h after the oral administration of the tested materials. The paw volume was measured with a micrometer screw gause at 1, 2, 3 and 4 h after the administration of the drug and the extract. The percentage inhibition of anti-inflammatory effect of the extract was calculated using the same formula for carrageenan-induced paw oedema.

### Statistical analysis

Data were presented as mean ± standard deviation (SD). Statistical analysis for the animal experiment was performed using one way analysis of variance (ANOVA) followed by Dunnett’s test with SPSS 11.5 software (Armonk, New York, USA). Any differences between the groups were considered significant at a level of *P* < 0.05.

## Results and discussion

### HPLC assay of bioactive polyphenols in *S. jambos*

**Fig. 1:**
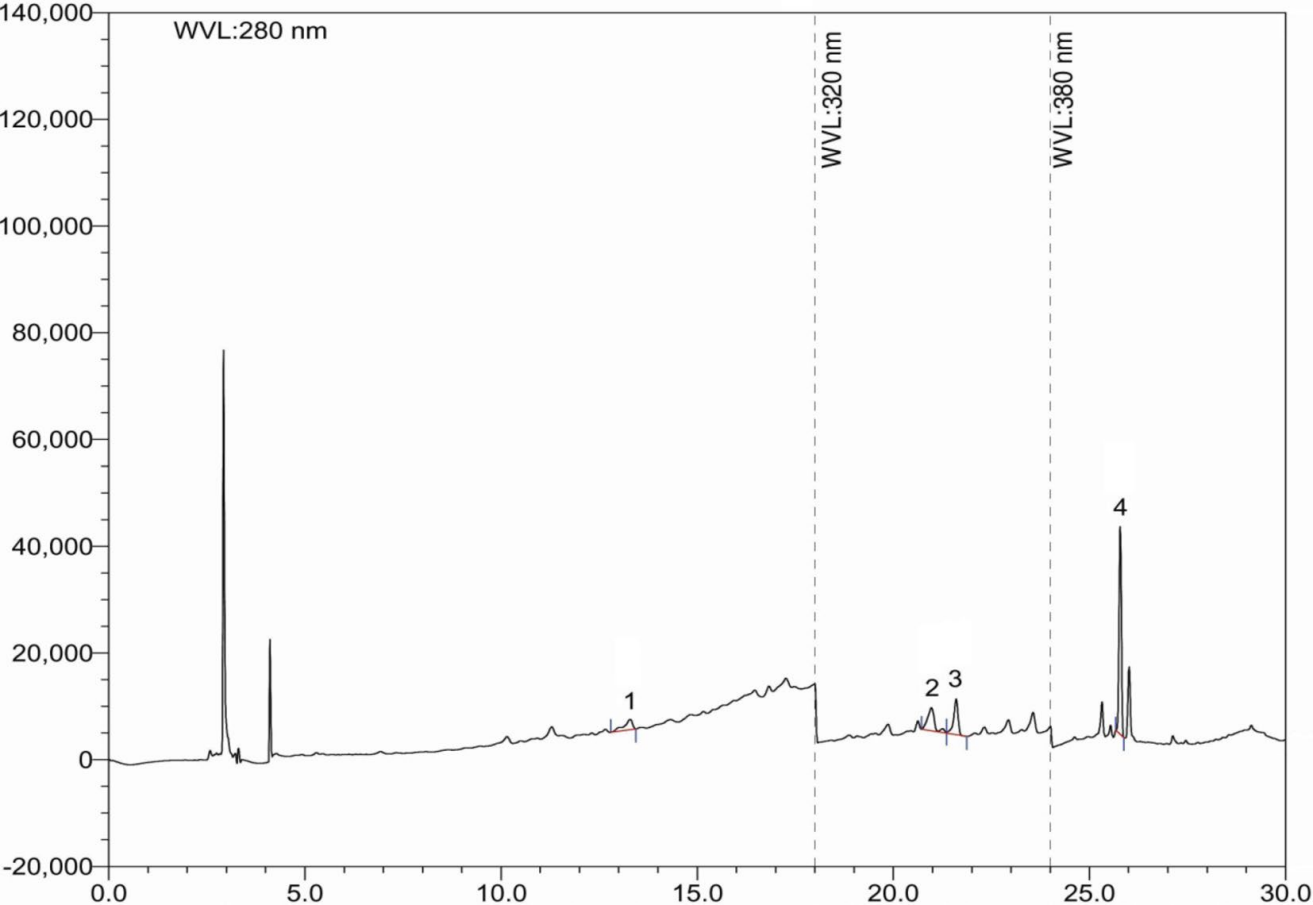
HPLC chromatogram of ethanol extract of *S. jambos*. *Peaks* *1* catechin hydrate (CH), *2* rutin hydrate (RH), *3* ellagic acid (EA), *4* quercetin (QU)

Individual phenolic compounds of *S. jambos* were identified and quantified by HPLC. The chromatographic separations of phenolic compounds in the ethanol extract are shown in Fig. 1. Based on the experimental results, of all the compounds present in *S. jambos*, catechin hydrate (99.00 mg/100 g of dry extract) was found to have the highest concentration followed by rutin hydrate at a slightly lower amount (79.20 mg/100 g of dry extract). Other compounds, such as ellagic acid and quercetin were also detected in moderate quantity (59.40, and 69.30 mg/100 g of dry extract, respectively). Table 1 demonstrates a clearer picture of the bioactive polyphenolic compounds present in the ethanol extract of *S. jambos*.

**Table 1:** Contents of bioactive polyphenolic compounds in the ethanol extract of *S. jambos *(n = 5)

Polyphenolic compound	Ethanol extract of *S. jambos*	% RSD
	Content (mg/100 g of dry extract)	
CH	99.00	1.88
RH	79.20	1.59
EA	59.40	1.36
QU	69.30	1.47

### Antioxidant activities

*S. jambos* was evaluated for its antioxidant activities. ABTS radical scavenging activity, reducing power assay, total antioxidant capacity, total phenolic content and total flavonoid content were followed for the antioxidant activity test.

The scavenging activity of ABTS was observed to increase with an increasing concentration (Table 2). At minimum concentration (100 µg/ml), the extract showed a better inhibition (97.82 %) than that of the standard ascorbic acid (95.58 %).The IC_50_ value of the extract was found to be significant (57.80 µg/ml), in comparison to that of the ascorbic acid (12.01 µg/ml). ABTS assay is generally used to evaluate the total antioxidant power of single compounds and complex mixtures of different plants [23]. The relative absorbance at 734 nm is used to evaluate the ABTS radical scavenging activity in both organic and aqueous solvents.

**Table 2:** ABTS radical scavenging activity of ethanol extract of *S. jambos* with ascorbic acid

Concentration (µg/ml)	% Inhibition at different concentration	
	Ethanol extract	Ascorbic acid
10	14.82 ± 0.23	48.60 ± 0.17
20	25.31 ± 0.12	85.79 ± 0.25
40	46.66 ± 0.19	99.19 ± 0.21
60	67.25 ± 0.13	99.25 ± 0.29
80	82.69 ± 0.16	99.53 ± 0.24
100	97.82 ± 0.15	95.58 ± 0.18
250	97.85 ± 0.14	99.85 ± 0.27
IC_50_	57.80 ± 0.18	12.01 ± 0.12

The values are expressed as mean ± standard deviation (n = 3)

The positive control used in determining the reducing power assay was ascorbic acid (Table 3). The maximum absorbance for *S. jambos* at 250 µg/ml was recorded to be 0.4934 µg/ml, while the standard ascorbic acid had given an absorbance of 1.111 µg/ml. The absorbance of the extract increased with an increase in the extract concentration. The hydrogen atom donated by the phenolic compounds aid in the conversion of Fe^3+^ to Fe^2+^. This in turn causes the free radical chain to break resulting in the reductones (antioxidants) to exert an antioxidant response [24]. The phenolic compounds present in *S. jambos* cause Fe^3+^/ferricyanide complex to reduce to its ferrous form (Fe^2+^), exhibiting reducing power ability.

**Table 3:** Reducing power assay of ethanol extract of *S. jambos* with ascorbic acid

Concentration (µg/ml)	Average absorbance at 700 nm	
	Ethanol extract	Ascorbic acid
10	0.0282 ± 0.014	0.3801 ± 0.012
20	0.0422 ± 0.016	0.4577 ± 0.017
40	0.0998 ± 0.017	0.5398 ± 0.023
60	0.1497 ± 0.013	0.6345 ± 0.037
80	0.1739 ± 0.021	0.7125 ± 0.013
100	0.2329 ± 0.014	0.7811 ± 0.029
250	0.4934 ± 0.027	1.1115 ± 0.009

The values are expressed as mean ± standard deviation (n = 3)

The total antioxidant capacity is determined using the phosphomolybdenum method. At an acid pH, the extracts cause Mo (VI) to gain an electron and reduce to Mo (V). This produces a green phosphate/Mo (V) complex. *S. jambos* showed significant antioxidant capacity. The total antioxidant capacity of the ethanol extract was found to be 628.5 mg ascorbic acid/g of extract (Table 4). The total antioxidant activity was found in relatively significant quantity when compared to the standard ascorbic acid per g of extract.

**Table 4:** Total antioxidant capacity of ethanol extract of *S. jambos*

Ethanol extract	Avg. absorbance at 695 nm	Total antioxidant capacity
		Mg of ascorbic acid equivalent (AAE) per gm of dry extract
*S. jambos*	0.329 ± 0.01	628.5 ± 0.14

The values are expressed as mean ± standard deviation (n = 3)

The total phenolic content calculated in *S. jambos* was quite high (230.82 mg/g of gallic acid equivalent). The total flavonoid content was also found in significant amount: 11.84 mg/g of quercetin equivalent per g of dry extract (Table 5). Presence of high concentration of phenolic compounds results in this high percentage inhibition value of the extract. The scavenging ability of the phenols is due to the hydroxyl groups in their chemical structure [25]. Polyphenolic compounds can inhibit mutagenesis and carcinogenesis in humans when ingested up to 1 g from a diet rich in fruits and vegetables on a daily basis [26]. Phytochemical components, especially polyphenols, such as flavonoids, phyenylpropanoids, phenolic acids, etc. are responsible for the free radical scavenging and antioxidant activities of plants. Certain flavonoids are also reported as potent free-radical scavengers [27–29]. Therefore, these phenolic compounds account for the significant antioxidant activity of *S. jambos.*

**Table 5:** Total phenolic and flavonoid content of ethanol extract of *S. jambos*

Ethanol extract	Total phenolic content	Total flavonoid content
	Mg of gallic acid equivalent (GAE) per g of dry extract	Mg of quercetin equivalent (QE) per gm of dry extract
*S. jambos*	230.82 ± 0.06	11.84 ± 0.01

The values are expressed as mean ± standard deviation (n = 3)

### Anti-inflammatory activity

#### Carrageenan-induced paw oedema

The anti-inflammatory effect of the *S. jambos* using carrageenan induced oedema tests is expressed in Table 6. The positive control (Indomethacin) significantly (P < 0.05; P < 0.01) reduced the paw oedema between the first and fifth hour once carrageenan was injected (53.06 to 62.94 % inhibition). Five hours after the carrageenan injection, a maximum oedema paw volume of 1.43 ± 0.25 mm was observed in the control group. Rats which received 400 mg/kg body weight of the extract were observed to significantly (P < 0.05) decrease (P < 0.05; P < 0.01) the carrageenan-induced oedema paw volume between the 1 and 5 h time interval, in comparison to that of the standard drug, indomethacin, at a dose of 10 mg/kg body weight. The inhibition percentage of the oedema paw volume at 1, 2, 3, 4 and 5 h by the 400 mg/kg body weight of *S. jambos* was also found to be higher relative to that by the indomethacin. The highest reduction in the paw volume by the 400 mg/kg body weight of the extract at 5 h was 58.04 %, while that by indomethacin was 62.94 %, respectively.

**Table 6:** Effects of ethanol extract of *S. jambos* and indomethacin on carrageenan-induced oedema paw volume in wistar rats

Treatment	Dose	Right hind paw volume (% inhibition)				
	(mg/kg)	1 h	2 h	3 h	4 h	5 h
Vehicle	10 (ml/kg)	0.98 ± 0.12	1.07 ± 0.16	1.26 ± 0.21	1.35 ± 0.23	1.43 ± 0.25
Indomethacin	10	0.46 ± 0.15 (53.06)*	0.47 ± 0.19 (56.07)*	0.53 ± 0.17 (57.94)*	0.54 ± 0.16 (60.00)*	0.53 ± 0.18 (62.94)**
*S. jambos*	200	0.74 ± 0.13 (24.49)*	0.69 ± 0.14 (35.51)*	0.74 ± 0.18 (41.27)**	0.75 ± 0.15 (44.44)*	0.73 ± 0.17 (48.95)**
*S. jambos*	400	0.65 ± 0.15 (33.67)*	0.58 ± 0.17 (45.79)**	0.59 ± 0.19 (53.18)**	0.61 ± 0.18 (54.82)*	0.60 ± 0.17 (58.04)**

Each value is presented as the mean ± SEM (n = 5)

* *P* < 0.05 compared with control group (Dunnett’s test)

** *P* < 0.01 compared with control group (Dunnett’s test)

Previous studies have found ethyl acetate and methanol extracts more effective, in comparison to 80 mg/kg of phenylbutazone [30]. However, in our study, it was found that at the dose of 400 mg/kg body weight (5 h), the ethanol extract of *S. jambos* grown in Bangladesh exhibited a significant activity in comparison to the standard drug, indomethacin (10 mg/kg body weight).

#### Histamine-induced paw oedema

Table 7 showed the anti-inflammation effect of the *S. jambos* using histamine-induced paw oedema tests. A maximum oedema paw volume of 1.52 ± 0.21 mm was observed in the control group 5 h after the histamine injection. Rats that were pre-treated with the extract at 400 mg/kg body weight significantly compressed (P < 0.05; P < 0.01) the histamine-induced oedema paw volume from 1 to 5 h, in comparison to that with the standard drug, indomethacin, at a dose of 10 mg/kg body weight. The percentage inhibition of the oedema paw volume at 1, 2, 3, 4 and 5 h by the 400 mg/kg body weight of the extract was also notably higher (P < 0.05; P < 0.01) than that by the indomethacin. The maximum reduction in the paw volume by the 400 mg/kg body weight of *S. jambos* at 5 h was 53.95 %, while that by the indomethacin went down to 65.79 %, respectively.

**Table 7:** Effect of ethanol extract of *S. jambos* and indomethacin on histamine-induced oedema paw volume in wistar rats

Treatment	Dose	Right hind paw volume (% Inhibition)				
	(mg/kg)	1 h	2 h	3 h	4 h	5 h
Vehicle	10 (ml/kg)	1.10 ± 0.15	1.25 ± 0.19	1.34 ± 0.18	1.40 ± 0.22	1.52 ± 0.21
Indomethacin	10	0.56 ± 0.14 (49.09)*	0.57 ± 0.16 (54.40)*	0.58 ± 0.15 (56.72)*	0.53 ± 0.19 (62.14)**	0.52 ± 0.13 (65.79)**
*S. jambos*	200	0.76 ± 0.11 (30.91)	0.80 ± 0.17 (36.00)**	0.86 ± 0.23 (35.82)*	0.84 ± 0.15 (40.00)**	0.82 ± 0.18 (46.05)**
*S. jambos*	400	0.67 ± 0.13 (39.09)	0.69 ± 0.14 (44.80)*	0.72 ± 0.16 (46.27)*	0.74 ± 0.14 (47.14)*	0.70 ± 0.19 (53.95)**

Each value is presented as the mean ± SEM (n = 5)

* *P* < 0.05 compared with control group (Dunnett’s test)

** *P* < 0.01 compared with control group (Dunnett’s test)

Carrageenan and histamine induced paw oedema were evaluated for their anti-inflammatory effect in *S. jambos*. The inflammatory response induced by carrageenan in rats is characterized by a biphasic response. These biphasic responses have marked oedema formation that results from the rapid production of several inflammatory mediators such as histamine, serotonin, and bradykinins. This is said to be the first-phase. Consequently, the second phase is the release of prostaglandins and nitric oxide with a peak at 3 h, which is produced by an inducible isoform of cyclooxygenase (COX-2) and nitric oxide synthase (iNOS) [31]. The present work has been an attempt to reduce the oedomatogenic response in rats evoked by carrageenan. From the results, it was found that pre-treated oral administration of the extract was effective in the reduction of the response. Henceforth, we can infer a relationship between the anti-inflammatory properties of the extract and the inhibition of intracellular signalling pathways in inflammatory mediators.

Histamine acts as an important inflammation mediator, potent vasodilator, and vascular permeability facilitator [32]. Rats were subcutaneously injected with histamine. The liquid spreads out in the body like a wheal. This results in an increase in the vascular permeability of the host capillary venules in the skin. Substances that inhibit the activity of histamine receptors shrink the area of the wheal formed. This could be because the anti-inflammatory activity of the extract is supported by its anti-histamine activity. The antihistaminic effect of the extract increases with the concentration of the extract. The extract inhibits the formation and action of the inflammatory mediators, effectively suppressing the production of oedema by histamine. This study shows that *S. jambos* possesses a greater anti-oedematogenic effect (P < 0.01) on paw oedema induced by carrageenan and histamine, in comparison to that of the standard drug (indomethacin) in treated rats. The pattern of anti-inflammatory activity exhibited by this extract might be similar to that of indomethacin.

HPLC analysis of the ethanol extract of *S. jambos* was used to determine the phenolic compounds responsible for the pharmacological activities and justify a correlation. Catechin and ellagic acid compounds are likely to play a role in the anti-inflammatory activity [33–35]. Furthermore, rutin hydrate and quercetin have also shown good anti-inflammatory activity [36–38]. HPLC studies confirm the presence of relatively high concentration of these antioxidant chemicals in *S. jambos*, which helps to explain the significant acute anti-inflammatory activity of this plant extract.

## Conclusions

The formation of oedema induced by carrageenan and histamine was greatly reduced by the ethanol extract of *S. jambos*, which explains the significant acute anti-inflammatory activity of the plant extract. Potential of *S. jambos* in anti-inflammatory and antioxidant activities could also possibly be due to the presence of bioactive polyphenolics compounds like, catechin hydrate, rutin hydrate, ellagic acid, and quercetin. The polyphenolic compound, catechin hydrate has been determined and reported for the first time ever. However, a more extensive study is necessary to determine the exact mechanism(s) of action of the extract.
